# Green biosourced composite for efficient reactive dye decontamination: immobilized *Gibberella fujikuroi* on maize tassel biomatrix

**DOI:** 10.1007/s11356-024-32809-w

**Published:** 2024-03-15

**Authors:** Sema Celik, Selen Kurtulus Tas, Fatih Sayin, Tamer Akar, Sibel Tunali Akar

**Affiliations:** 1 Department of Chemistry, Faculty of Science, Eskisehir Osmangazi University, 26040 Eskisehir, Turkey; 2 Department of Chemistry, Graduate School of Natural and Applied Sciences, Eskisehir Osmangazi University, 26040 Eskisehir, Turkey

**Keywords:** Azo dye, Biosorption, Breakthrough, Fungal cell, Immobilization, Maize tassel, Reactive Yellow 2 (RY2), Wastewater treatment

## Abstract

Biosorptive treatment with microbial biomass is regarded as an environmentally friendly and effective way to reduce dye contamination in contaminated aquatic environments. Immobilizing microbial cells for use in this process can significantly improve their effectiveness as biosorbents in the water treatment process. The current investigation searches for a sustainable and environmentally friendly approach to decolorization by employing a green biocomposite material sorbent system (ZM@GFC) created by immobilizing fungal cells (*Gibberella fujikuroi*) on maize tassel tissues to efficiently remove Reactive Yellow 2 (RY2) from contaminated water sources. Batch and dynamic flow tests were performed to evaluate the biodecolorization properties of the newly created immobilized biomaterial as well as the effects of several essential operating conditions factors on the sorption behavior. Biosorption yields of 95.7% and 90.0% in batch and dynamic modes were achieved for experimental dye decolorization. The biosorption of RY2 by ZM@GFC occurred fast and achieved equilibrium within 60 min. The pseudo-second-order kinetic model elucidated the dye biosorption onto ZM@GFC. The Langmuir model provided a more accurate representation of the results than the Freundlich model. At the same time, Redlich-Peterson isotherm demonstrated the best level of agreement with the experimental data. These findings indicate that the biosorption mechanism predominantly involved the formation of a monolayer covering and that the energy properties of the ZM@GFC surface were uniform. The breakthrough capacity at the exhaustion time was 537.32 mg g^−1^. The predicted cost of generating ZM@GFC was anticipated to be 61.03 USD/kg. The investigations on safe disposal demonstrated that the biosorption process did not generate any secondary pollution. In conclusion, using maize tassel tissue as an immobilized decolorization agent offers a possible method for removing reactive azo dye pollutants from the aquatic medium that is both economical and environmentally benign.

**Supplementary Information:**

The online version contains supplementary material available at 10.1007/s11356-024-32809-w.

## Introduction

Increasing urbanization has caused significant amounts of industrial effluents to be dumped into the environment in recent years. Dyes, heavy metals, antibiotics, insecticides, nano-wastes, microplastics, and other organic and inorganic pollutants are introduced into resources (Foroutan et al. 2022, Lee and Tang 2020, Liu et al. 2008, Ma et al. 2022, Moslehi et al. 2024, Yaashikaa and Kumar 2022). Dyes are among the most problematic wastewaters to treat owing to a small percentage of color in water, which is reason for worry as it is evident and alters the aesthetic value, water clarity, and gas solubility in aquatic sources (Bulgariu et al. 2019, Parihar and Malaviya 2023). Besides affecting the environment, the hideous consequences of dyes include skin irritation upon touch, an irritant to the respiratory system, renal failure, blindness, carcinogenicity, and the production of male bladder cancer (Jathanna et al. 2023; Salmani et al. 2016; Siddiqui et al. 2018).

Numerous physico-chemical and decontamination procedures, including nanofiltration and reverse osmosis, electrocoagulation, advanced oxidation, and photo-catalysis, are costly and limited in scope (Bensalah et al. 2023; Kumar et al. 2023). As a result, developing affordable biomaterials for the removal of dye from wastewater has received much attention. Biosorption of dye using fungus has been the topic of several investigations as a potential, cost-effective, and environmentally friendly alternative to traditional wastewater treatment (Sahithya et al. 2022). Many successful strategies, such as immobilization, have been utilized to change microbial cells, with immobilization of cells being an important step in the bioremediation process. Carrier materials such as polymers, natural substances, magnetic nanoparticles (MNPs), and inorganic–organic materials have been extensively investigated in this field (Akar et al. 2013; Bouabidi et al. 2019; Gong et al. 2022).

In this research, a novel immobilized material was created to remove color from dye-containing water. Maize (*Zea mays,* ZM) tassel tissues served as a carrier material for the immobilized *Gibberella fujikuroi* (*G*. *fujikuroi*, GF) cells, and the target pollutant was Reactive Yellow 2 (RY2), an azo dye. To the best of our knowledge, there have been no prior investigations reported in the literature regarding the biodecolorization capability of this biomaterial. The biodecolorization features of the recently developed biocomposite material (ZM@GFC) were exhaustively investigated using batch and dynamic flow tests by examining the effects of the mean experimental conditions on the dye removal. Interactions between RY2 molecules and ZM@GFC were evaluated by several kinetic and isotherm models. In addition, breakthrough curve studies of ZM@GFC were investigated. Finally, experiments on the RY2 removal from synthetic wastewater were also conducted to test the practical applicability of ZM@GFC.

## Material and methods

### Materials

The cob of corn was purchased from the market, and the tassel tissues under the leaves were separated. Silky parts were repeatedly washed with deionized water and dried at 60 ℃ in an oven. Reactive Yellow 2 (RY2) (C_25_H_15_C_l3_N_9_Na_3_O_10_S_3_) was chosen as the model contaminant and was purchased from Merck. Stock dye solution (1000 mg L^−1^) was used to prepare the solutions with different concentrations (25 to 600 mg L^−1^) by fresh dilution before the biosorption experiments. pH of the RY2 solutions was adjusted between 2 and 10 using 0.1 N NaOH (Sigma-Aldrich, ≥ 97%) and 0.1 N HCl (Sigma-Aldrich, 37%) solutions.

### Preparation of biocomposite sorbent system

*G*. *fujikuroi* was routinely maintained at 4 °C on potato dextrose agar (PDA) slants. Cells were cultured in the autoclaved liquid medium (at 121 ℃) for 7 days. The constituents of the liquid growing medium contain (per liter) glucose (8.0 g), NH_4_NO_3_ (2.4 g), KH_2_PO_4_ (5.0 g), MgSO_4_.7H_2_O (0.82 g), and a solution containing trace elements (2.0 mL). This medium’s pH was adjusted to 5.0. The composition of trace elements solution (per liter) are as follows: ZnSO_4_·7H_2_O (0.50 g), C_6_H_8_O_7_·H_2_O (0.50 g), (NH_4_)_2_Fe(SO_4_)_2_·6H_2_O (0.10 g), CuSO_4_·5H_2_O (0.025 g), MnSO_4_·H_2_O (0.005 g), Na_2_MoO_4_·2H_2_O (0.005 g), and H_3_BO_3_ (0.005 g) (de Oliveira et al. 2007). All these chemicals were ACS grade (≥ 99%) and purchased from Sigma-Aldrich.

For the passive immobilization process, 1 g of dried maize tassel tissue as matrix material was added into 100 mL of liquid growing medium in the Erlenmeyer before the sterilization step. Sterilization was performed in an autoclave at 121 °C for 15 min. One milliliter of *G*. *fujikuroi* cell suspension was added to the cooled Erlenmeyer under aseptic conditions and incubated in an orbital shaker at 25 ℃ and 120 rpm for 7 days. At the end of the incubation period, the biocomposite was separated by filtration from the growing medium, washed three times with distilled water, and dried overnight in an oven at 60 °C. This immobilized biocomposite was ground, sieved using a 212-μm sieve, and stored in a glass flask.

### Batch studies

Batch system experiments were performed by optimizing parameters such as pH, biosorbent dosage, time, and initial concentration of dye. A definite amount of immobilized biocomposite was added into 25 mL of dye solution and stirred on a multi-magnetic stirrer at 300 rpm. After the biosorption process, suspensions in the beaker were centrifuged at 5000 rpm for 5 min to separate the biosorbent from the aqueous medium.

The effect of initial pH on RY2 decolorization was examined in the pH range of 2–10. pH adjustment was made by adding appropriate amounts of HCl and NaOH solutions into 25 mL of RY2 solutions. Biosorption experiments were performed using 0.4–4 g L^−1^ of maize silk, *G*. *fujikuroi*, and ZM@GFC to determine the optimum biosorbent dosage. Contact time for the RY2 biosorption onto ZM@GFC was changed in the time range of 5–90 min. The initial RY2 concentration was varied from 25 to 600 mg L^−1^ to evaluate the biosorption isotherms. To investigate the effect of salt on the decolorization potential of ZM@GFC, KCl solution changing between 0.02 and 0.15 M was added into the dye solution of 100 mg L^−1^ and the optimized biosorption procedure was carried out. The effectiveness of ZM@GFC for RY2 decolorization at real conditions was assessed using simulated wastewater at optimum batch conditions (Guo et al. 2008).

### Column studies

Flow mode biosorption experiments were performed using glass columns at 25 ℃. The multi-channel peristaltic pump was used for the continuous flow system, and columns were connected to the pump through Tygon tubing. The columns were packed with accurately weighted (0.08, 0.1, 0.12, 0.15, 0.18, 0.2, and 0.25 g) ZM@GFC, and RY2 solutions at pH 2.0 were pumped downflow. The flow rate was varied between 0.5 and 6.0 mL min^−1^ to obtain maximum RY2 removal.

ZM@GFC-loaded fixed-bed column’s breakthrough and exhaustion points and capacities were identified through breakthrough trials. In these experiments, RY2 solutions (100 mg·L^−1^) were passed through ZM@GFC in the fixed-bed column (internal diameter, 1.05 cm) using a downflow mode while maintaining specific conditions (flow rate = 2.0 mL·min^−1^, ZM@GFC amount = 80 mg). Following the end of the biosorption process, the effluents were collected and analyzed. Equation (1) (Aksu and Gönen 2004) was used to calculate the biosorption column’s capacity of ZM@GFC in terms of mg·g^−1^ at exhaustion time (*t*_ex_, min), where *q*_ex_ was the exhaustion capacity of the ZM@GFC loaded to biosorption column, *V*_ex_ (mL) is the volume of RY2 solution that passed downward through the column until *t*_ex_, and *C*_in_ and *C*_ef_ (mg·L^−1^) are initial and final RY2 concentrations, respectively. *m* represents ZM@GFC mass (g) used in the biosorption column:

1 $${q}_{ex}={\int }_{0}^{{V}_{ex}}\frac{{C}_{in}-{C}_{ef}}{m}dV$$

Equations (2) and (3) can be utilized to determine the total effluent (dye) volume (*V*_ef_, mL) and the quantity of RY2 that biosorbed onto ZM@GFC (*m*_ad_, g). *F* represents the flow rate in mL min^−1^.

2 $${V}_{{\text{ef}}}=F{\times t}_{{\text{ex}}}$$

3 $${m}_{{\text{ad}}}={\int }_{0}^{{V}_{{\text{ex}}}}{(C}_{{\text{in}}}-{C}_{{\text{ef}}}) dV$$

To determine the total percentage of decolorization (*R*, %) and the length of the mass transfer zone (*Z*_m_, mm), Eqs. (4) and (5) (Aksu and Gönen 2004) can also be employed:

4 $$R=\frac{{m}_{{\text{ad}}}}{{C}_{{\text{in}}}\times {t}_{{\text{ex}}}\times F}\times {10}^{5}$$

5 $${Z}_{{\text{m}}}=H\times \left(1-\frac{{t}_{br}}{{t}_{ex}}\right)$$

where *H* represents the bed height in millimeters.

The Thomas, Bohart-Adams (B–A), and Yoon-Nelson (Y–N) models were commonly used for kinetic investigations of breakthrough curve modeling. Nonetheless, three of these models are analogous and can be regarded as mathematically equivalent, as shown in the simplified equation (Eq. (6)) suggested by Chu (Chu 2020). The relevant model parameters (*a* and *b*) are described in supplementary materials (Table S1).

6 $$\frac{{{\text{C}}}_{{\text{ef}}}}{{{\text{C}}}_{{\text{in}}}}=\frac{1}{1+{\text{exp}}\left(a-bt\right)}$$

RY2 concentrations in the solutions were specified using a UV/vis spectrophotometer (Shimadzu UV 2550) at *λ*_max_ value for RY2 dye (404 nm). Biosorption yield (%) and biosorbed RY2 concentration (*q*_e_, mg g^−1^) were determined using Eqs. (7) and (8), respectively.

7 $$\mathrm{Biosorption yield}=\frac{{C}_{{\text{o}}}-{C}_{{\text{e}}}}{{C}_{{\text{o}}}}\times 100$$

8 $$q=\frac{{C}_{{\text{o}}}-{C}_{{\text{e}}}}{m}\times V$$

where *C*_0_ and *C*_e_ are the initial and equilibrium RY2 concentrations in milligrams per liter, *m* is the quantity of ZM@GFC in g, and *V* is the volume of the RY2 solution in liter.

### Characterization

ZM@GFC was characterized using FTIR spectroscopy to determine the functional groups in the structure of the biosorbent. FTIR spectra were taken before and after RY2 biosorption. To understand the surface structure of ZM@GFC, unloaded and RY2-loaded biocomposite sorbents were visualized by scanning electron microscopy (SEM). Zeta potential analysis was performed to determine the surface charge density of the biosorbent.

## Results and discussion

### Effect of pH on the decolorization

**Fig. 1:**
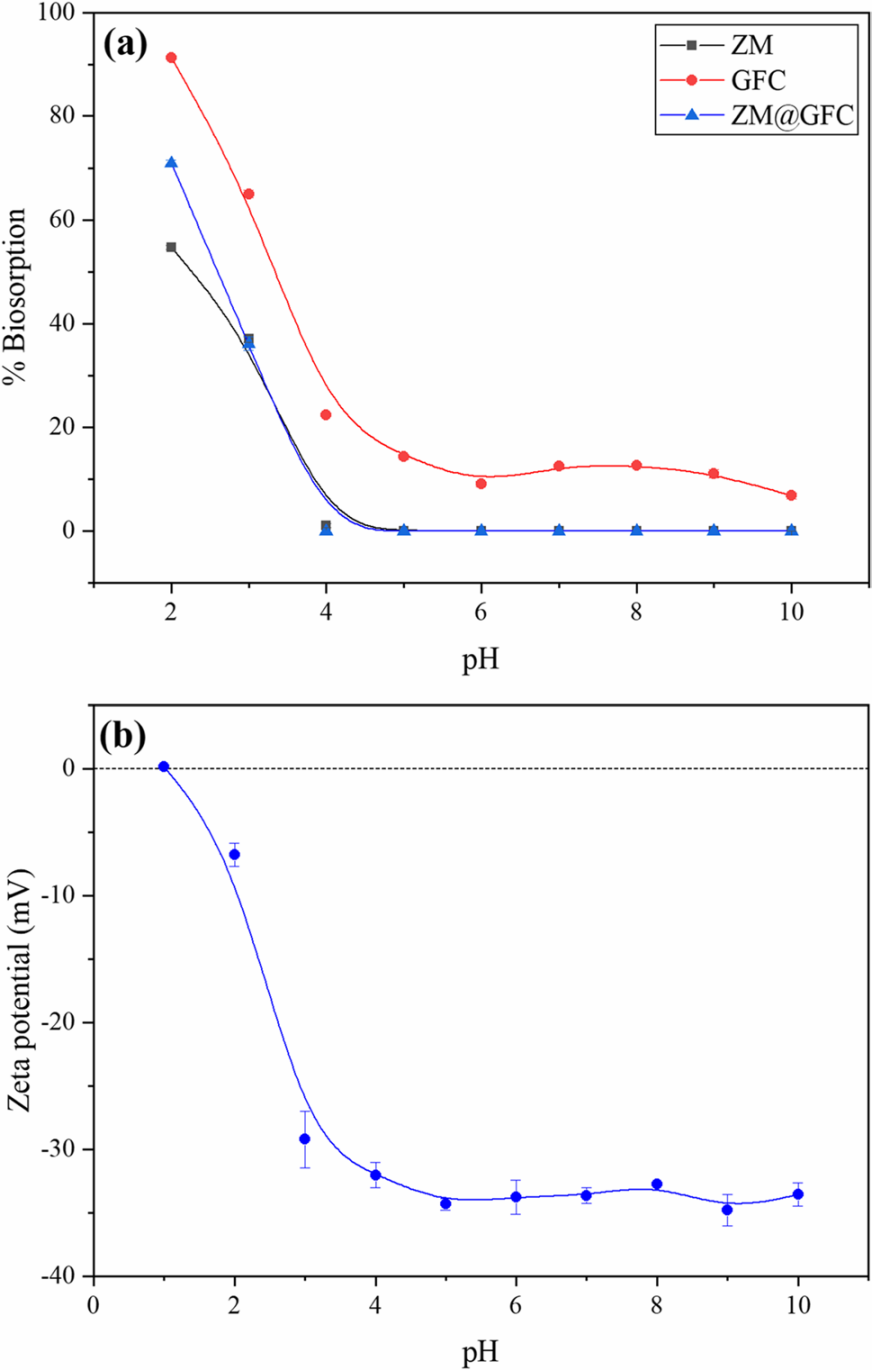
**a** Effect of pH for RY2 decolorization on ZM, GFC, and ZM@GFC. **b** Zeta potentials of ZM@GFC at various pH values

The most important factor controlling the biosorptive decolorization studies is the initial pH of the sorption medium. It can alter the sorbent material’s surface charge. As a result, depending on the organic dye’s anionic or cationic nature and how it interacts with the surface of the sorbent material, the sorption efficiency will either reduce or increase with rising solution pH (Arab et al. 2022). Due to their structure’s sulfonate group(s), reactive dyes ionize at high levels in the aqueous phase to produce colored anions (Akar and Celik 2011). RY2 dye molecule contains three sulfonate groups. Accordingly, decolorization experiments were carried out using the ZM@GFC dosage of 2.0 g L^−1^ and an initial RY2 concentration of 100 mg L^−1^ over a pH range of 2.0–10.0. Figure 1a illustrates the dependence of the biosorptive decolorization efficiencies of ZM@GFC on pH level. The pH value has a substantial impact on the decolorization efficiencies of ZM, GFC, and ZM@GFC, as shown in Fig. 1. High decolorization efficiency was observed at pH 2.0 in this figure. With the initial pH of the dye solution rising from 2.0 to 4.0, it was observed that ZM@GFC’s decolorization efficiency decreased proportionally, and at pH 5.0 and higher, no color was removed. The electrostatic attractive forces between the anionic RY2 molecules and the protonated binding sites of the ZM@GFC surface cause the finding of the maximum decolorization efficiency at pH 2.0. While the number of negatively charged binding sites on the ZM@GFC surface increased with increasing pH, the number of protonated binding sites decreased. Deprotonation, which happens on its surface as pH increases, can be used to describe this change. The zeta potential analysis of the ZM@GFC also verified the optimal pH value for the decolorization process. ZM@GFC’s isoelectric point (IEP) was determined to be 1.0 (Fig. 1b). The high proton concentration in the medium produces a strong positive surface charge on the biomaterial at a pH lower than the IEP. Anionic RY2 molecules can thus readily interact with surfaces of positively charged biomaterial. These findings are also in line with the earlier studies that used pH-responsive biomaterials to decolorize reactive dye-contaminated solutions (Akar and Celik 2011, Iqbal and Saeed 2007, Sayin 2022).

### Effect of ZM@GFC dosage

**Fig. 2:**
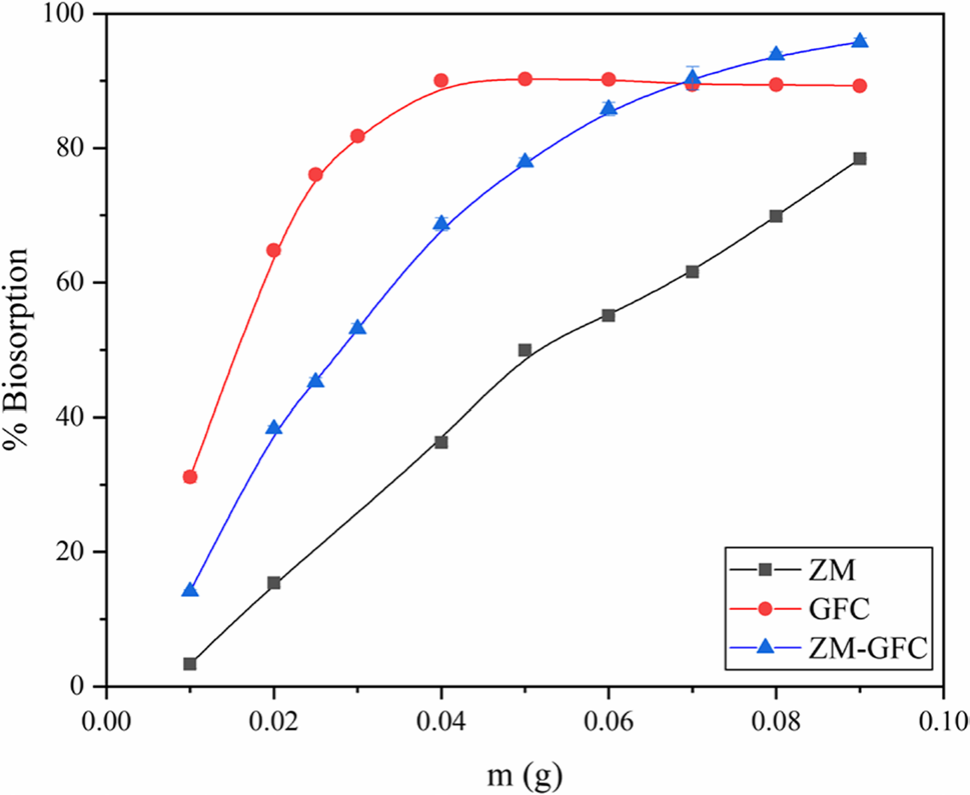
Effect of biosorbent amount for RY2 decolorization on ZM, GFC, and ZM@GFC

The right dosage of the biomaterial is one of the crucial factors that must be established for the practical applicability of a suggested biomaterial for decolorization. As seen in Fig. 2, biomaterial dosage significantly impacted the uptake of RY2 dye by ZM, GFC, and ZM@GFC, and the decolorization increased considerably as the biomaterial dose increased until saturation at a specific value. With increasing doses of the biomaterial, the decolorization yields of GFC and ZM@GFC increased and nearly stabilized at 0.05 g (2.0 g L^−1^) and 0.08 g (3.2 g L^−1^) of biomaterials, respectively. The rising trend in decolorization was mediated by the increased number of active functional sites available for the biosorption of dye molecules. The ensuing consistent decolorization tendencies of the biosorbents are most likely caused by dye molecules occupying active sorption sites on the surfaces of the biomaterials. Other studies about the biosorption of synthetic dye molecules onto *Saccharomyces cerevisiae* (Mahmoud 2016), malt bagasse (Juchen et al. 2018), and *Rhizopus nigricans* emphasized similar findings (Kumari and Abraham 2007).

ZM@GFC was selected for further studies among the sorbent materials examined in this study because of its significant capabilities. *Zea mays*, a natural waste that can be acquired for no cost, is a component of this biomaterial. So, the cost of the treatment procedure will also be lower when using biocomposite sorbent for dye removal. Therefore, a dosage of 0.08 g (3.2 g L^−1^) for immobilized biosorbent (ZM@GFC) was chosen for the following decolorization assays.

### Kinetics of the RY2 decolorization process

**Fig. 3:**
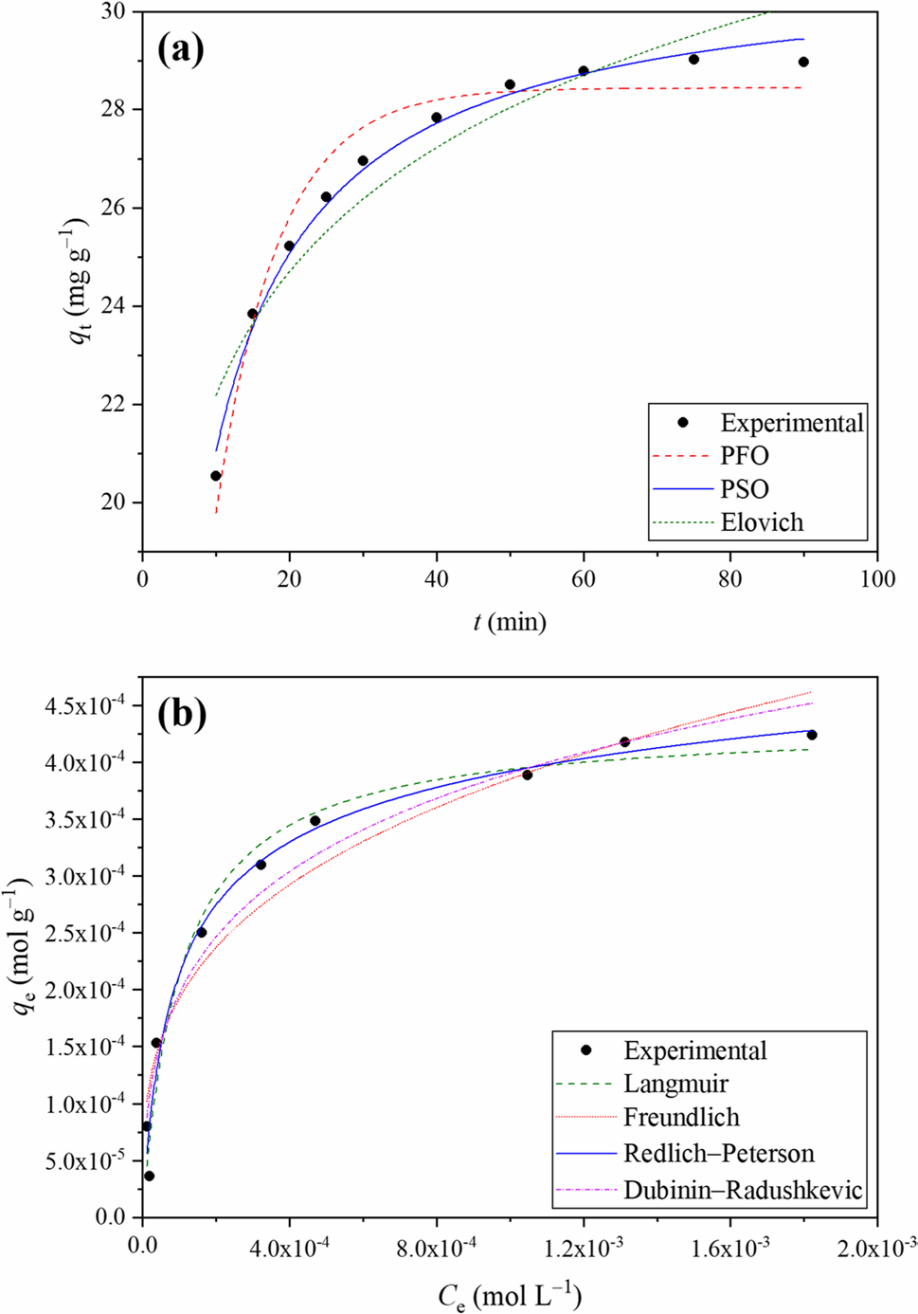
**a** Experimental data and the kinetic curves for RY2 decolorization by ZM@GFC. **b** Experimental data and the isotherm curves for RY2 decolorization by ZM@GFC

The sorption kinetics was investigated to ascertain the time-sensitivity of dye removal and sorption mechanism. Figure 3a shows the kinetic profile of the proposed biocomposite sorbent for the removal of RY2 contamination. The figure shows that the ZM@GFC’s biosorption capacity grew swiftly during the initial stages of decolorization before settling into equilibrium with a relatively slow biosorption rate. At 60 min, ZM@GFC’s biosorption capacity reached its maximum level. The substantial concentration gradient between solution and sorption sites on the surface of the sorbent and the easily available active binding sites are both responsible for the rapid initial phase (Elwakeel and Al-Bogami 2018). In the subsequent slow period, a decrease was observed in this concentration gradient. The RY2 uptake on ZM@GFC after 60 min of contact time was 95.7% (30.34 mg g^−1^). The practical use and efficacy of the biosorbent materials depend significantly on fast sorption kinetics and a short equilibrium period. In order to identify responsible mechanisms governing biosorption dynamics, experimental data were applied to nonlinear forms of the Lagergren pseudo-first-order (Lagergren 1898), the pseudo-second-order (Ho and McKay 1999), and Elovich model (Zeldowitsch 1934) equations, represented by Eq. (9), (10), and (11), respectively:

9 $${q}_{{\text{t}}}={q}_{{\text{e}}}\cdot \left(1-{\text{exp}}\left(-{k}_{1}t\right)\right)$$

10 $${\text{q}}_{\text{t}}\text{=}\left(\frac{{\text{k}}_{2}{\cdot {\text{q}}}_{\text{e}}^{2}\cdot {\text{t}}}{\text{1} + {\text{k}}_{2}\cdot {\text{q}}_{\text{e}}^{1}\cdot {\text{t}}}\right)$$

11 $${q}_{t}=\frac{1}{\beta }{\text{ln}}\left(1+\alpha \cdot \beta \cdot t\right)$$

Figure 3a also displays the fitting of the Lagergren pseudo-first-order, the-pseudo-second-order, and Elovich models to the data from the experiments. Table 1 reports the kinetic parameters derived from these kinetic models. According to its higher coefficient of determination value among the applied models, the pseudo-second-order model was in greater conformity with the empirical findings. Additionally, as shown in Table 1, the equilibrium sorption capacities derived from the pseudo-second-order model (*q*_e_, calc) are notably similar to the obtained experimentally (*q*_e_, exp). These details showed that the pseudo-second-order model could offer a more accurate depiction of the entire biosorption dynamic. The results also suggest that the biosorptive decolorization of RY2 contamination may have a rate-limiting phase called chemisorption. Such a good fit of the pseudo-second-order was reported by Radwan et al. (2020) (modified *Chlorella vulgaris* sorption on Reactive Yellow 145), da Silva and Pietrobelli (2019) (chia seeds sorption on Reactive Yellow B2R), and Temesgen et al. (2018) (banana and orange peels sorption on Reactive Red Dye).

**Table 1:** Kinetic model constants for biosorption of RY2 on ZM@GFC

Pseudo-first-order				Pseudo-second-order				Elovich			
*k*_1_	*q*_*e*_	*r*^2^	χ^2^	*k*_2_	*q*_*e*_	*r*^2^	χ^2^	*α*	*β*	*r*^2^	χ^2^
(min^−1^)	(mg g^−1^)			(g mg^−1^ min^−1^)	(mg g^−1^)			(mg g^−1^ min^−1^)	(g mg^−1^)		
1.19 × 10^−1^	28.44	0.957	0.36	6.84 × 10^−3^	30.99	0.990	0.09	159.46	0.274	0.906	0.80

### Isotherm models fitting of the RY2 decolorization process

The relationship between the pollutant in the aqueous phase at a given temperature and the pollutant sorbed from the aqueous phase onto a given sorbent material is known as the sorption isotherm. The sorption isotherms are necessary to estimate the sorption mechanism, the sorbent material’s surface properties, and other factors affecting the sorption process. The creation of a sorption isotherm is a crucial first stage in the design of a sorption process scheme (Al-Mhyawi et al. 2023).

The decolorization potential of ZM@GFC for RY2 dye was examined in this investigation using the Freundlich, Langmuir, Redlich–Peterson, and Dubinin–Radushkevich isotherms. According to the Freundlich model (Freundlich 1906), sorption takes place on a heterogeneous surface with minimal interaction between the adsorbed molecules. The Langmuir model (Langmuir 1918) implies that effluent removal from the aqueous phase takes place on homogeneous surfaces via monolayer sorption with no interactions among sorbed molecules. The Redlich–Peterson isotherm model (Redlich and Peterson 1959) integrates the properties of both these models across a broad range of effluent concentrations. At lower concentrations (α ~ 1), the Redlich–Peterson equation approximates the Langmuir model, whereas at higher concentrations (α ~ 0), it approximates the Freundlich model. This concept is applicable to both types (heterogeneous and homogeneous) of processes (Foo and Hameed 2010). The Dubinin–Radushkevich (Dubinin and Radushkevich 1947) model is used for determining the free energy of the biosorption, and this model is temperature-dependent.

The non-linear mathematical expressions of these isotherm models can be stated as:

12 $$\begin{array}{cc}{\text{Langmuir}}:& {q}_{{\text{e}}}=\frac{{q}_{{\text{m}}}\cdot {K}_{{\text{L}}}\cdot {C}_{{\text{e}}}}{1+{K}_{{\text{L}}}\cdot {C}_{{\text{e}}}}\end{array}$$

13 $$\begin{array}{cc}{\text{Freundlich}}:& {q}_{\text{e}}={K}_{{\text{F}}}\cdot {C}_{\text{e}}^{1/{\text{n}}}\end{array}$$

14 $$\begin{array}{cc}{\text{Dubinin}}-{\text{Radushkevich}}:& {q}_{{\text{e}}}={q}_{{\text{m}}}\cdot {\text{exp}}\left(-\beta \cdot {\varepsilon }^{2}\right)\end{array}$$

15 $$\begin{array}{cc}{\text{Redlich}}-{\text{Peterson}}:& {q}_{e}=\frac{{K}_{{\text{R}}}{C}_{{\text{e}}}}{1+{{\text{a}}}_{{\text{R}}}{C}_{{\text{e}}}^{\mathrm{\alpha }}}\end{array}$$

where the equilibrium concentration of RY2 molecules and the equilibrium sorption capacity in solution, respectively, are denoted by *C*_e_ (mol L^−1^) and *q*_e_ (mol g^−1^). The Freundlich constant is *K*_F_ ((mol g^−1^) (mol L^−1^))^1/n^, and the heterogeneity factor is 1/*n*. *K*_L_ is the Langmuir constant related to the sorption energy (L mol^−1^), and *q*_max_ is the maximal monolayer sorption capacity (mol g^−1^) of the sorbent material. *ε* represents the Polanyi potential (*ε* = *RT* ln (1 + 1/*C*_e_), and *E* represents the free energy (*E* = 1/√(2*β*)) of the biosorption of RY2 by ZM@GFC (kJ mol^−1^). Redlich–Peterson constants are *a*_R_ (mg L^−1^) and *K*_R_ (L mol^−1^).

As shown in Fig. 3b, the biosorptive capacity increases 3.62 × 10^−5^ mol g^−1^ to 4.34 × 10^−4^ mol g^−1^ along with the initial RY2 concentration 25 mg L^−1^ to 600 mg L^−1^. This increase occurs due to the more potent driving force provided by the higher concentration gradient, which overcomes the mass transfer resistances to dye diffusion towards the biosorption sites (Bayat et al. 2023). Figure 3b also indicated the fitted isotherm models to experimental data. The constant parameters and coefficient of determination values for the isotherm models are shown in Table 2. The Redlich–Peterson isotherm with the highest *r*^2^ and lowest Chi-square (χ^2^) showed the best fit at the investigated temperature. The RY2 biosorption process consists of many different mechanisms that combine homogeneous monolayer and heterogeneous biosorption conditions (Aksu 2002). α ~ 1 indicates that the Langmuir model described the experimental findings more accurately than the Freundlich model (Liu et al. 2010). This model signified that the biosorption mechanism generally acted as monolayer coverage and that the ZM@GFC surface is energetically homogenous. The results reflect that the RY2 sorption capacity of ZM@GFC (90.0 mg g^−1^) is comparable (Table 3) to that of different waste-based biosorbent materials used for RY2 removal (Celik et al. 2021; Karagöz et al. 2018; Tunali Akar et al. 2016a; Won et al. 2016) (Table 4).

**Table 2:** Isotherm model constants for biosorption of RY2 on ZM-GFC

Langmuir				Freundlich				Redlich-Peterson				Dubinin-Radushkevich			
*q*_m_	*K*_L_	*r*^2^	χ^2^	*n*	*K*_F_	*r*^2^	χ^2^	*α*_R_	*K*_RP_	*r*^2^	χ^2^	*q*_*m*_	*E*	*r*^2^	χ^2^
(mol g^−1^)	(L mol^−1^)				((mol g^−1^) (mol L^−1^)^−1/n^)			(L mg^−1^)	(L mol^−1^)			(mol g^−1^)	(kJ mol^−1^)		
4.35 × 10^−4^	9.59 × 10^3^	0.974	6.2 × 10^−10^	3.32	3.08 × 10^−3^	0.931	1.7 × 10^–9^	0.90	5.95	0.982	5.1 × 10^−10^	9.44 × 10^−1^	12.84	0.956	1.1 × 10^−9^

**Table 3:** RY2 biosorption capacities of different sorbents from the literature

Biosorbent	*q*_max_ (mg g^−1^)	Reference
Leaf tissue immobilized with microorganisms	51.1	(Celik et al. 2021)
Magnetic *Lactarius salmonicolor* cells	115.2	(Karagöz et al. 2018)
*Corynebacterium glutamicum*	104.0	(Won et al. 2016)
Sugar beet pulp	62.9	(Tunali Akar et al. 2016a)
Immobilized *G. fujikuroi* on maize tassel biomatrix	90.0	This work

**Table 4:** Column parameters for RY2 biosorption onto ZM@GFC

*V*_eff_	*q*_br_	*q*_ex_	*m*_ad_	*R*%	*Z*_m_	*k*_BA_, *k*_T_	*N*_o_	*q*_T_	*k*_YN_	τ	*r*^2^
(mL)	(mg g^−1^)	(mg g^−1^)	(mg)	(%)	(cm)	(mL mg^−1^ min^−1^)	(mg L^−1^)	(mg g^−1^)	(min^−1^)	(min)	
655	4.82	537.32	42.99	27.02	0.29	0.0802	23.71	513.23	0.0297	203.9	0.977

### Salt effect and wastewater applications

**Fig. 4:**
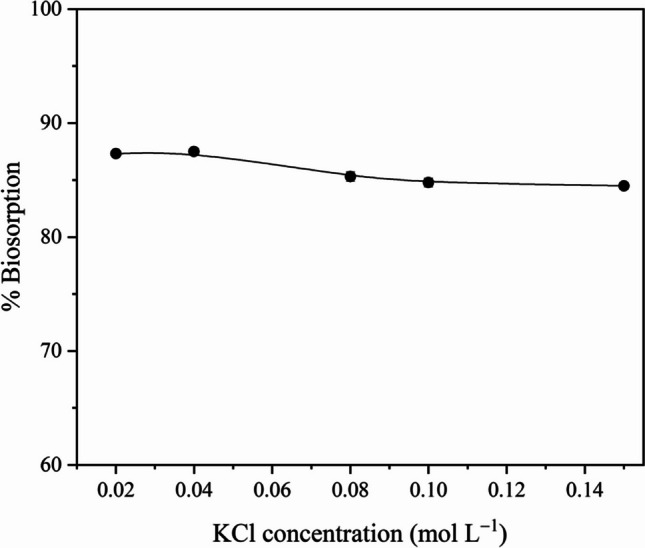
Effect of KCl concentration on RY2 decolorization on ZM@GFC

RY2 decolorization experiments on ZM@GFC were performed in solutions containing KCl at different concentrations ranging from 0.02 to 0.2 mol L^−1^ to determine the impact of the salt effect. RY2 decolorization yield of ZM@GFC remained nearly constant when KCl content in the biosorption medium was increased (Fig. 4). 2.8% of the biosorption yield was lost when the KCl concentration was increased from 0.02 to 0.2 mol L^−1^. This result means that most of its decolorization performance is maintained even in conditions with high salt concentrations. At optimum batch biosorption circumstances, RY2 had a biosorption yield of 95.7% for ZM@GFC in synthetic wastewater added with 100 mg L^−1^ of dye. This finding indicates the practical use of the proposed immobilized biosorbent.

### Column studies

**Fig. 5:**
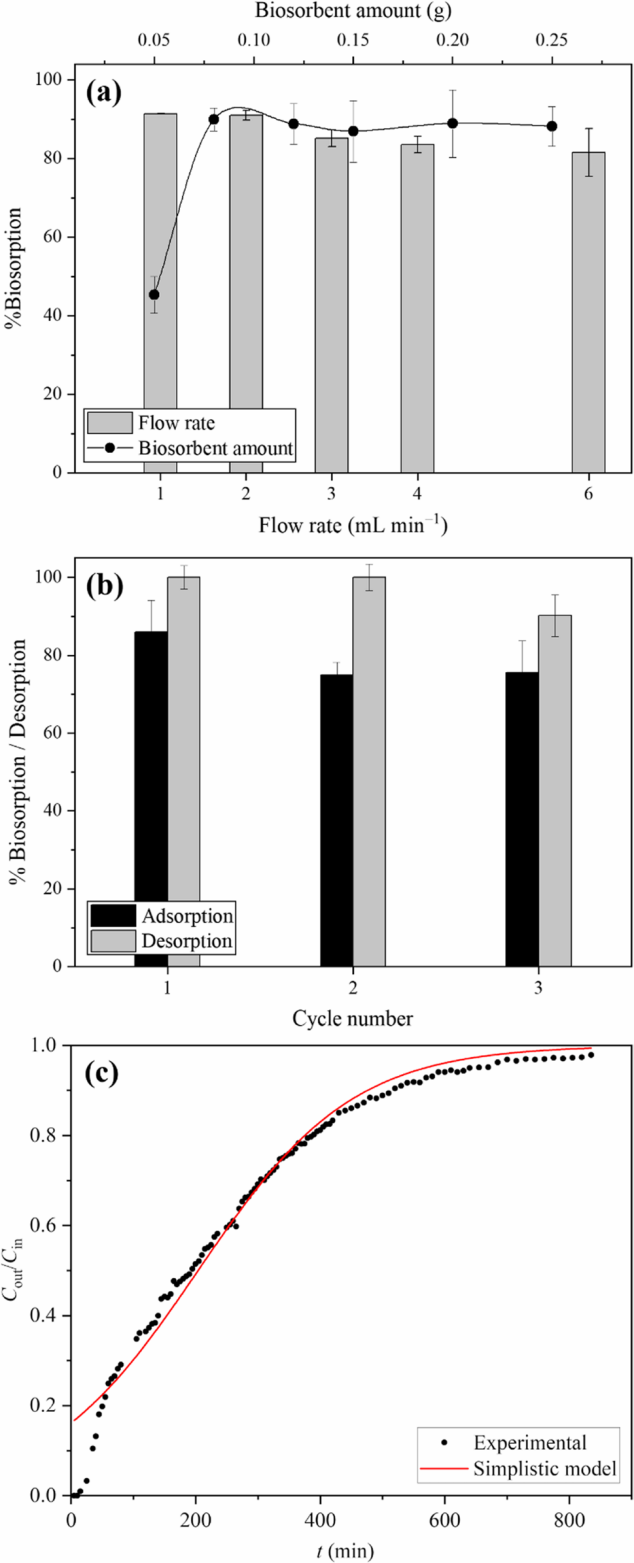
**a** Effect of flow rate and biosorbent amount on RY2 decolorization, **b** biosorption and desorption cycles of RY2, and **c** breakthrough curve for RY2 biosorption on ZM@GFC

The column technique is one of the most frequent ways to use biosorbents in dye-contaminated wastewater treatment. The primary factors tested were the flow rate and biosorbent mass packed in the columns (Fig. 5a). The percentage RY2 removal yield of ZM@GFC rose from 81.6 to 91.0% (*p* < 0.05) when the flow rate was reduced from 6.0 to 2.0 mL min^−1^. A saturation point was reached after the definite flow rate due to insufficient time for residency at higher flow rates (El Messaoudi et al. 2016). As a result, the investigations of the dynamic flow were performed at a flow rate of 2.0 mL min^−1^. However, increasing the quantity of ZM@GFC packed into the column from 0.05 to 0.08 g resulted in an increase in the RY2 decolorization yield of ZM@GFC from 45.3 to 90.0% (Tunali Akar et al. 2016b). Figure 5a shows that the decolorization yield of ZM@GFC increased very little (*p* > 0.05) when the ZM@GFC dose was increased from 0.08 to 0.25 g. This finding could be explained by the fact that RY2 binding sites on ZM@GFC reached their maximum capacity. Therefore, 0.08 g of ZM@GFC was packed into the column for further research in dynamic flow mode.

The desorption potential of ZM@GFC was investigated by 0.01 M NaOH in the fixed bed column at preoptimized dynamic flow conditions. RY2 solution with a concentration of 100 mg L^−1^ was supplied at the onset of each sorption cycle. After every desorption cycle, the bed was washed with water at 2.0 mL min^−1^. Figure 5b shows that ZM@GFC could be readily regenerated using an acidic solution, exhibiting a remarkably high desorption rate (~ 100%) throughout the first and second cycles. The adsorption efficacy of the adsorbent reduced from 86 to 76% in the further cycles. This could be explained by the persistent occupation of some active sites on ZM@GFC by the RY2 molecules or deterioration of the ZM@GFC surface under intense alkaline environments (Sultana et al. 2022).

### Breakthrough curve modeling

The breakthrough curve for RY2 biosorption (Fig. 5c) obtained by graphing *C*_f_/*C*_in_ at *t* (min) was used to assess the biosorption efficiency, and Table 3 contains the predicted parameters derived from this dynamic biosorption study. As expected, until *t*_br_ (*C*_f_ = 0.1*C*_in_), a greater RY2 biosorption was noticed at the start of column operation (25 min). The observed pattern could be attributed to the initial availability of fresh ZM@GFC, which led to greater biosorption, followed by 50% breakthrough (195 min) and exhaustion (*C*_f_ = 0.9*C*_in_) time (520 min). When the saturation capacity values in the recent studies using a biosorption fixed-bed column design are compared, it is clear that ZM@GFC (537.32 mg g^−1^) could effectively remove reactive dye from wastewater; sewage-sludge-based biochar, 42.30 mg g^−1^ (Al-Mahbashi et al. 2022); sulfuric acid activated red mud, 106 mg g^−1^ (Mavinkattimath et al. 2023), *Thamnidium elegans* immobilized on *Phragmites australis*, 104.58 mg g^−1^ (Sayin 2022); *Neurospora sitophila* immobilized *Platanus orientalis* leaf, 50.09 mg g^−1^ (Celik et al. 2021); and chitosan-oxalic acid-biochar composite, 160 mg g^−1^ (Doondani et al. 2022).

Bohart-Adams, Yoon-Nelson, and Thomas models were also applied to data obtained from breakthrough experiments by non-linear regression modeling (Eq. 6), and the results are presented in Fig. 5c and Table 3. Coefficients of determination (*r*^2^) in this table proved that the computed and experimental data were in excellent agreement, indicating that the equation is valid for RY2 biosorption. The compatibility between the experimental saturation capacity and the capacity value (*q*_T_) calculated using the Thomas model indicated that the prepared biosorbent could be used effectively for RY2 biosorption in the fixed-bed columns at large-scale investigations. Furthermore, the experimental 50% breakthrough time is consistent with the parameter (τ) in the Yoon-Nelson model.

### Characterization of ZM@GFC

**Fig. 6:**
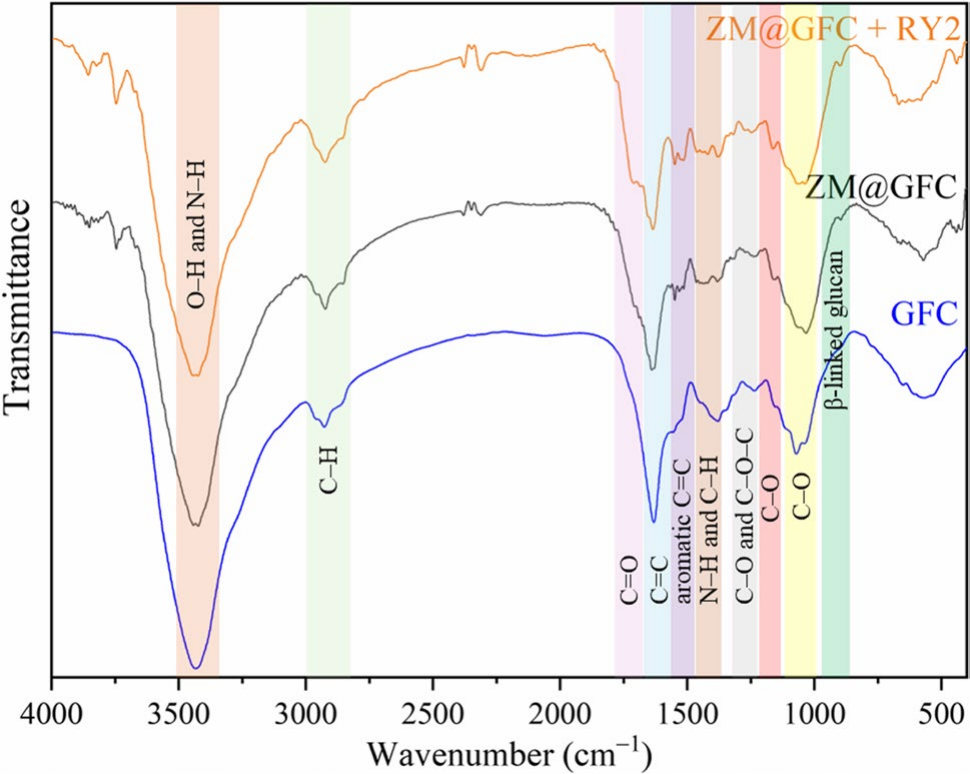
FTIR spectra of ZM@GFC and RY2 biosorbed ZM@GFC

The prominent absorption bands in the FTIR spectra of GFC and ZM@GFC (Fig. 6) were due to the stretching vibrations of –OH groups covered by N–H stretching vibrations (about 3400 cm^−1^, broadband), C–H stretching vibrations of alkane and methylene groups (2924 and 2855 cm^−1^), C═O stretching vibrations of the carboxylic group (1707 cm^−1^), C═C stretching vibrations of aromatic alkenes (1639 cm^−1^), and C─O stretching vibrations of aliphatic groups (1032 cm^−1^). New absorption bands at 1549 cm^−1^ and 899 cm^−1^ were observed in the spectrum of biocomposite when compared with the FTIR spectrum of fungal biomass. These bands can be ascribed to aromatic C = C groups and *β*-linked glucan structure of lignocellulosic matrix material (Huang et al. 2018). These similar stretching vibrations were also reported in FTIR spectrum of *Zea mays* biomass (Devi et al. 2022; Ekinci 2023; Jia et al. 2020). A comparison of spectra, before and after RY2 biosorption, exhibits minimal shifting in all these absorption bands.

**Fig. 7:**
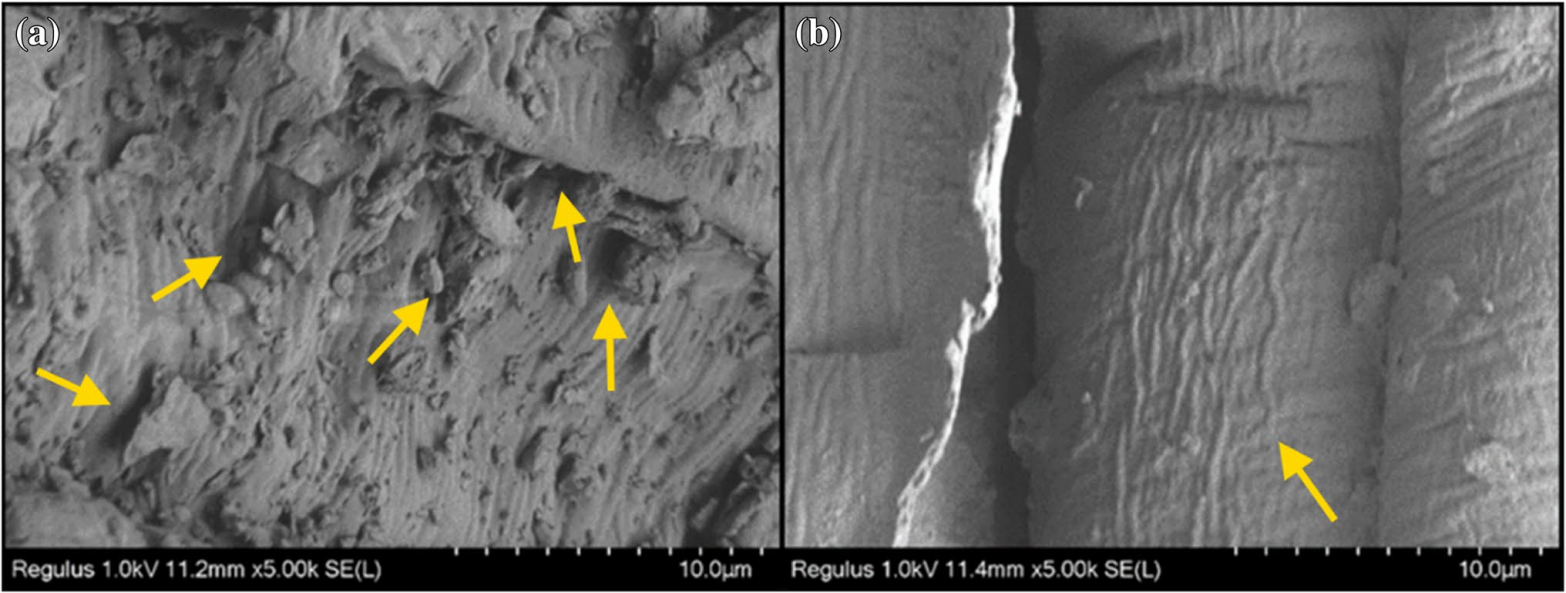
SEM micrographs of ZM@GFC (**a**) and RY2 biosorbed ZM@GFC (**b**)

The intensities of the bands at 1549, 1514, and 1420 cm^−1^ related to amine (N–H) groups (Weißpflog et al. 2021) and 1381 cm^−1^ related to C─H bending vibration increased after the biosorption of RY2 onto ZM@GFC. The absorption bands at 1381 cm^−1^ (C─H bending) (Akar et al. 2021), 1238 cm^−1^ (C–O/C–O–C stretching or CH_2_ wagging) (Huang et al. 2018; Zhang et al. 2011), and 1155 cm^–1^ (stretching vibrations of C–O of alcohols) (Liu et al. 2022) also shifted to 1379, 1248, and 1161 cm^−1^, respectively, after the dye biosorption process. These results confirmed the biosorption of RY2 at all the mentioned binding sites on the ZM@GFC surface. SEM micrographs depicted the morphology of ZM-GFC (Fig. 7a) and RY2-loaded ZM@GFC (Fig. 7b). The micrographs show that ZM@GFC has a diverse surface structure with a variety of holes, bumps, and grooves. The immobilized biomaterial’s unique surface enables RY2 molecules to be easily impregnated onto its surface, enabling interaction with the dye binding sites present. After the RY2 biosorption process onto ZM@GFC, it becomes apparent that the surface of the biomaterial appears tighter and smoother. This finding supports the notion that the dye has successfully been biosorbed to the surface of ZM@GFC.

### Mechanism of the biosorption process

**Fig. 8:**
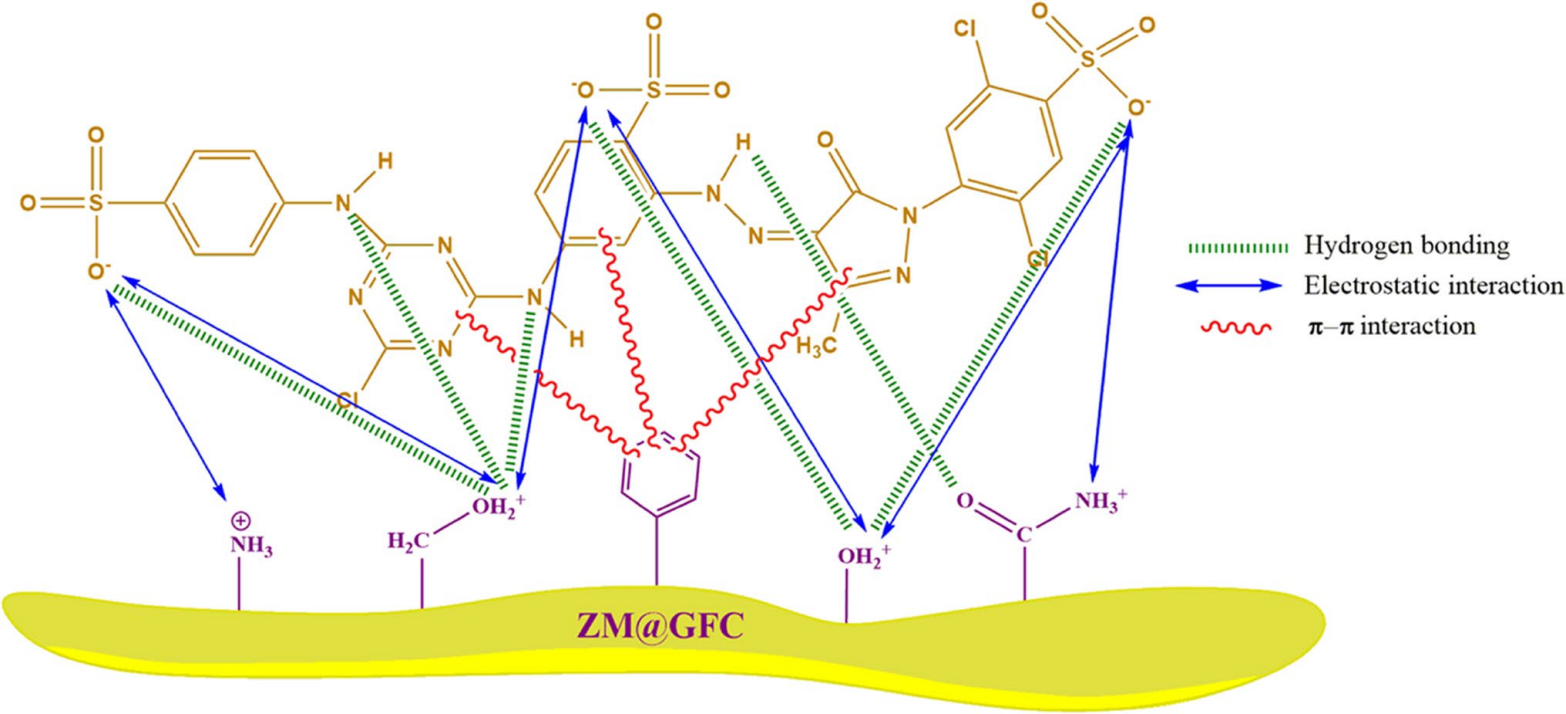
Possible interaction mechanisms between ZM@GFC and RY2

The pH-depended biosorption profile revealed that electrostatic interaction between ZM@GFC surface and RY2 anions may play a significant role in the dye removal process. The fungal biomass and lignocellulosic matrix material provide potential functional groups for the removal of RY2. In this context, H-bonding and π-π interactions are conceivable due to the presence of π electrons in the components and RY2 molecules (Ghosh et al. 2022; Nag et al. 2020). A visual representation of the biosorption process, H-bonding, π-π interaction, and electrostatic interaction of RY2 by ZM@GFC is shown in Fig. 8.

### Safe disposal of used biosorbent

Environmental safety is a crucial concern when it comes to disposing of the biosorbent that contains dye effluents. To prevent the release of RY2 and the subsequent contamination, the utilized biosorbent was subjected to incineration at a temperature of 800 ℃ for 1 h. Ash sample of 0.08 g (optimum sorbent amount) was treated with 25 mL deionized water by mixing for 24 h. Supernatant was spectroscopically analyzed to determine the dye concentration in the liquid phase (Ghosh et al. 2022; Nag et al.^2019^). The results indicated that no RY2 dye was detected in the leachate. This finding proved that the spent biosorbent could be eliminated by combustion without generating any secondary pollutants from the biosorption process. This form of the spent material could be safely stored and utilized in clay brick production units in rural areas, interred in landfills, or applied to road construction (Ghosh et al. 2021).

### Cost estimation of the biosorbent

The cost of producing the biosorbent is the most important factor determining the economic viability of biosorption process. The use of a low-cost matrix material in immobilization reduces the overall sorbent cost and provides a biocomposite with higher sorption potential. Furthermore, reusability feature of ZM@GFC is another important factor influencing the overall process cost. The total production cost of 1 kg of ZM@GFC is calculated to be approximately 61.03 USD which includes the cost of all chemicals (31.51 USD), power (21.52 USD), and raw materials (8.00 USD). Considering that commercial activated carbon (Carl Roth) price (~ 140 USD/kg), the suggested biosorbent could be used as an economic and eco-friendly alternative for water treatment processes (Banerjee et al. 2018; Celik et al. 2021; Tunali Akar et al. 2022).

## Conclusion

In this research, *G*. *fujikuroi* cells passively immobilized on maize tassel tissues showed promising results for RY2 decolorization. Both batch and continuous bioremoval procedures achieved a target dye output of over 90%. The Redlich-Peterson isotherm and the pseudo-second-order kinetic model were used to make sense of the results of the dye biosorption by ZM@GFC. SEM, FTIR, and zeta potential analysis provided insight into the biodecolorization process of ZM@GFC. Encouraging results were also obtained from integrating the suggested material into dynamic flow mode applications, in addition to successful batch applications. According to the breakthrough curve study, the fixed-bed column packed with ZM@GFC reached its exhaust 520 min. Overall, the results of the current investigation indicate that the proposed new biocomposite could serve as an efficient, affordable, sustainable, and environmentally friendly option for treating waters contaminated with reactive dyes.

### Supplementary Information

Below is the link to the electronic supplementary material.

Supplementary file1 (DOCX 24 KB)

## Data Availability

The data used in this study are available from the corresponding author upon request.
