# Supplementary material for: Green biosourced composite for efficient reactive dye decontamination: immobilized Gibberella fujikuroi on maize tassel biomatrix
Source: Environ Sci Pollut Res Int. 2024 Mar 15;31(17):25836–48. doi: 10.1007/s11356-024-32809-w (PMC11023956; doi:10.1007/s11356-024-32809-w)
Supplement: Supplementary file 1 — Supplementary file1 (DOCX 24 KB) [file 11356_2024_32809_MOESM1_ESM.docx]

**Supplementary Information**

**Green biosourced composite for efficient reactive dye decontamination: Immobilized *Gibberella fujikuroi* on maize tassel biomatrix**

**Sema Celik ^1,^*, Selen Kurtulus Tas^2^,  Fatih Sayin^1^, Tamer Akar ^1^, Sibel Tunali Akar^1^**

*^1^ Department of Chemistry, Faculty of Science, Eskisehir Osmangazi University, 26040, Eskisehir, Turkey*

*^2^ Department of Chemistry, Graduate School of Natural and Applied Sciences, Eskisehir Osmangazi University, 26040, Eskisehir, Turkey*

**Corresponding author:* Sema Celik

*E–mail address:* secelik@ogu.edu.tr

Tel: +90–222–2393750/2870

Fax: +90–222–2393578

**Table S1.** B–A, Thomas, and Y–N model parameters in terms of Chu's simplified equation

| Model |  | B–A | Thomas | Y–N |
| --- | --- | --- | --- | --- |
| Parameters | *a* | *k*_BA_ *N*_o_ *L* / *u* | *k*_T_ *q*_T_ *m* / *Q* | k_YN_ τ |
|  | *b* | k_BA_ C_in_ | k_T_ C_in_ | k_YN_ |

*k*_BA_: B−A rate constant (mL/mg min), *L*: ZM-GFC height (cm), *N*_o_: B−A saturation concentration (mg/L), *u*: superficial velocity (cm/min), *k*_T_: Thomas kinetic rate constant (mL/mg min), *q*_T_: equilibrium sorption capacity calculated from Thomas model, *k*_YN_: Y−N kinetic constant (1/min), *Q*: flow rate (mL/min), and *τ*: 50% breakthrough time (min),
